# Supplementary material for: Are carbon emissions trading and green financial instruments synergistic? -Comprehensive quantitative research based on content analysis
Source: PLoS One. 2024 Mar 7;19(3):e0298601. doi: 10.1371/journal.pone.0298601 (PMC10919601; doi:10.1371/journal.pone.0298601)
Supplement: S1 Data — (ZIP) [file pone.0298601.s001.zip › Data/地级市Z_F工作报告.zip!/║■─╧/╥μ╤⌠╩╨/└┤╫╘╣1⁄2╓┌║┼í╛┬φ┐╦╩2╛▌═°í┐_2014.docx]

各位代表：

　　现在，我代表市人民政府向大会作政府工作报告，请予审议，并请市政协委员和列席会议的同志提出意见。

　　2013年工作回顾

　　2013年，全市人民在中共益阳市委的坚强领导下，团结拼搏，务实奋进，实现了经济的平稳较快发展和社会大局的和谐稳定。全年完成生产总值1130亿元（预计数，下同），比上年增长11%；完成公共财政总收入86亿元，增长14.6%；完成固定资产投资844亿元，增长35%；实现社会消费品零售总额402亿元，增长13.5%；城乡居民人均可支配收入11167元。年末金融机构本外币各项存款余额1012亿元，比年初增加150亿元；各项贷款余额524亿元，比年初增加100亿元。

　　（一）三次产业协调发展。三次产业结构比由上年的20:45:35调整为18.4:45.4:36.2。工业经济较快增长。全市完成工业增加值484亿元，比上年增长12.4%。新进规模工业企业84家，全市规模工业企业达866家，完成规模工业增加值452亿元，增长13%。高新技术企业实现增加值118亿元，增长30%。农业生产稳步发展。全市实现农林牧渔业增加值207亿元，比上年增长2.6%。粮食生产保持稳定，蔬菜、黑茶、养殖等产业健康发展。农产品加工业实现增加值126亿元，增长13.7%。抗御了50年一遇的特大旱灾。第三产业不断提升。第三产业实现增加值409亿元，比上年增长13.2%。交通运输、房地产、旅游、服务外包、文化创意等服务业发展势头较好。

　　（二）“两大会战”和“双百工程”强力实施。交通建设大会战扎实推进。全年完成交通基础设施建设投资81亿元，比上年增长15.6%。岳常高速益阳段等项目建成通车，资阳大桥全线贯通，石长铁路复线益阳段、二广高速益阳段、G319益阳南线高速等项目全面推进。益娄高速启动征地拆迁，益南高速、益安高速等项目对接和前期工作加快推进。水上交通运输秩序整治成效明显。园区建设大会战取得实效。全市九大园区完成基础设施建设投资116亿元，标准化厂房竣工面积130万平方米，新开工工业项目140个，新投产工业项目244个，均超额完成目标任务。“双百工程”顺利启动。市里研究确定了对全市经济社会发展具有重大战略意义与重要支撑作用的百件大事、百个重大项目，并已发布实施，“大干事、干大事”的氛围逐渐浓厚。扎实开展重点工程建设“三项行动”，全市重点调度的130个项目完成投资237.6亿元，为年度投资计划的102.5%。

　　（三）城乡统筹扎实推进。城镇建设步伐加快。规划编制工作得到拓展，规划执法得到强化。完成城镇基础设施建设投资63.3亿元。中心城区完成投资39.7亿元，市政项目建设扎实推进，完成了第五个“十万株树进城”任务。以创建国家卫生城市为载体，强力推进市容秩序、环境卫生等8大专项整治，创卫工作顺利通过了国家暗访和技术评估。创建国家交通管理模范城市工作顺利推进。农村基础设施建设不断加强。实施水利“十大工程”，完成水利建设投入20亿元，完成各类水利工程5.9万处。完成农村公路建设601公里，完成危桥改造、渡改桥45座。完成了100个行政村的农网升级改造。新增通宽带行政村120个。统筹城乡“八项规定动作”完成较好。农村土地流转面积达282万亩，其中耕地184万亩，耕地流转率全省第一。25个农民集中居住区建设顺利推进。市县统筹城乡专项规划全部编制完成。其他规定动作取得初步成效。

　　（四）改革开放不断深化。各项改革不断深化。行政审批制度改革深入推进，市本级实施的行政许可项目由274项精简至98项。国库集中收付制度改革、公务卡结算改革和预算绩效管理改革稳步推进。全市国有企业改革任务基本完成。教育、医疗卫生、水务、人事改革等有序推进。要素保障能力增强。益阳农商银行挂牌开业。新增小额贷款公司4家。益阳高新区成功发行15亿元企业债券。全年批回各类建设用地17055亩，有效保障了合理用地需求。共争取上级财政性资金154.7亿元，比上年增长20.4%。对外开放成效明显。全市招商引资实际利用市域外资金355亿元，完成固定资产投资283.5亿元，比上年增长24.8%。其中，直接利用外资1.75亿美元，增长22.4%；利用境内省外资金190亿元，增长18.2%。我市与蒙古国布尔干省、哈萨克斯坦乌卡市正式结为友好城市，与长三角、珠三角、港澳地区的交流与合作进一步加强。加快发展对外贸易，全市完成进出口总额4.8亿美元，比上年增长36%，其中出口4.3亿美元，增长38%。完成加工贸易进出口额8000万美元，比上年增长40%。

　　（五）绿色发展取得成效。节能减排扎实推进。全市万元生产总值能耗下降4%，万元规模工业增加值能耗下降5%；实施各类减排项目86个，全市102家重点污染源稳定达到国家排放标准，主要污染物减排完成省定目标。环境保护和生态建设得到加强。实施了十大环保工程项目，扎实推进了志溪河、兰溪河综合整治，加强了重金属污染防治和矿山地质环境治理，加大了农村环境综合整治力度。巩固国家森林城市创建成果，大力推进高效生态林业建设，完成造林24.5万亩，森林覆盖率稳定在54.4%。两型示范建设取得新的成效。10个单位被评定为省级两型示范创建单位。益阳东部新区两型示范区基础设施和项目建设取得新的进展。

　　（六）民生民利持续改善。民生得到新的改善。全市新增城镇就业3.5万人，新增农村劳动力转移就业3.07万人，城镇登记失业率控制在4.3%以内。推进了人力资源和社会保障基层公共服务平台建设，启动了社会保障卡发放，全市五大社会保险新增参保2.5万人。实现了“新农合”和城镇居民医保机构的整合。城乡低保月人均补助标准首次达到全省平均水平，五保供养水平进一步提高。改扩（新）建乡镇敬老院12所。落实农村义务教育经费保障机制改革资金2.85亿元，学生75.9万人次受益。落实低收入群体价费优惠政策，减轻群众负担5000多万元。全市基本建成保障性住房5.2万余套。中心城区棚户区改造稳步推进。完成特困移民避险搬迁安置946户3487人。省政府下达的21项实事30个指标均完成或超额完成。社会事业全面发展。争取国家和省级科技项目91个。7个项目获省科技进步奖。实现专利授权1060件，其中有效发明专利240件。加大教育投入，维修和改扩建校舍面积72万平方米，建成了75所合格学校和18所农村公办幼儿园。校车安全管理工作得到加强。建立健全全民医保体系，巩固完善国家基本药物制度，公共卫生服务均等化水平得到提高，重大疾病防控取得实效。医疗质量与安全管理得到强化。食品药品安全监管力度不断加大，食品药品领域安全形势良好。巩固和发展文化体制改革成果，“益阳文博现象”和“益阳花鼓戏现象”内外反响热烈。国务院公布益阳7处国家重点文物保护单位，铁铺岭重大考古发现引起各界关注。首届中国（湖南）花鼓文化艺术节成功举办。人口计生工作得到加强，低生育水平保持稳定。全民健身运动蓬勃开展，107个行政村安装了体育健身器材。全市新闻媒体的舆论导向和宣传影响不断增强。残疾人“两个体系”建设加快实施。突出加强价格调控，市场物价总体平稳。加强土地和矿产资源管理，连续14年实现耕地占补平衡。民兵组织调整改革全面完成，征兵工作改革顺利实施，国防动员组织指挥能力稳步提高，国防教育得到加强，人民防空建设扎实推进。优抚安置工作落到实处，双拥工作扎实开展。统计、审计、档案、保密、台办、科协、地方志、红十字、消防、民族宗教、地震、气象、水文、住房公积金、无线电管理、老龄等工作取得新成绩。工会、共青团、妇联等群团组织及各民主党派、工商联、无党派人士在经济社会发展中发挥了积极作用。

　　（七）社会大局和谐稳定。精神文明建设深入推进。开展了全市第三届道德模范评选活动，群众性精神文明创建和未成年人思想道德建设扎实推进，志愿者（义工）服务工作取得实效。民主法治建设不断加强。大力推进法治益阳建设，狠抓“法治县（市、区）”和“依法办事示范窗口单位”创建活动，普法宣传教育有序开展。行政执法公信力得到提升。加强法律援助工作，各级援助中心受理法律援助案件1503件。安全生产形势稳定好转。严格按照“四个确保”的目标，狠抓安全生产责任落实和措施落实，全市生产经营性事故和死亡人数比上年分别下降10.7%和35.7%。平安益阳创建扎实开展。突出强基固本，社会治安形势保持稳定。落实维稳工作第一责任，推行“五个特别工作法”，创新群众工作机制，扎实抓好矛盾纠纷排查调处，成功处置了一批突发事件，有效应对各种网络舆情，保持了社会大局的持续稳定。

　　（八）“三访三化”成效明显。全市创造性的开展了以“领导接访实效化，干部下访规范化，党员走访常态化”为主要内容的“三访三化促发展解民忧”专项活动。活动开展以来，全市共有1500多个工作组、9.6万多名党员干部参加“三访三化”活动，下到村组和社区，走访群众、了解民意、倾听呼声，先后收集和分类整理各方面的问题1.3万多个，已协调和解决9800多个，得到了基层和群众的认可，受到了权威媒体和国家有关部委的关注和肯定。

　　（九）政府自身建设得到加强。加强民本政府建设，以“12345市长热线”、信访接待等为主平台，着力解决了全市人民关心关注的“十大热点问题”和一大批事关群众切身利益的实际问题。自觉接受人大及其常委会的法律监督、工作监督，充分吸纳和利用政协在协商民主建设中的重要成果，认真办理人大代表建议和政协委员提案。全面推进政务公开、网上政务服务和绩效评估工作，服务效能得到提升。严格执行中央、省、市有关作风建设规定，落实廉政建设责任制，规范行政权力运行，推进“三公”经费公开，开展政府专项资金财务监察，加强招投标监管，深化工程建设领域专项治理，严肃查处违法违纪案件，努力营造风清气正的良好政务环境。

　　各位代表，一年的成绩来之不易，成绩的取得，是中共益阳市委正确领导的结果，是全市人民同心同德、开拓奋进的结果。在此，我代表市人民政府，向全市广大干部群众，向各位人大代表、政协委员，向离退休老同志，向各民主党派、工商联、无党派人士和各人民团体，向中央和省属驻益单位，向驻益人民解放军、武警官兵和公安干警，向所有关心、支持和参与益阳改革发展的各界朋友，表示衷心的感谢！

　　在看到成绩的同时，我们也清醒地认识到，当前我市经济社会发展中还存在不少困难和问题。主要是：发展速度不快，生产总值等增速没有达到年初目标；经济总量不大，发展质量不高，扩总量、转方式、调结构的任务非常艰巨；改革开放的任务十分繁重，力度需要进一步加大；发展环境需要进一步优化，等等。对这些问题，我们将采取有力措施，切实加以解决。

　　2014年形势把握和改革发展要求

　　2014年是全面贯彻落实党的十八届三中全会精神的重要一年，是实现“十二五”目标的关键一年。从全球来看，2014年世界经济仍将延续缓慢复苏态势，但也存在不稳定不确定因素，新的增长动力源尚不明朗，大国货币政策、贸易投资格局、大宗商品价格的变化方向都存在不确定性。从国内来看，党的十八届三中全会胜利召开，吹响了全面深化改革的号角。中央将继续坚持稳中求进工作总基调，保持宏观经济政策连续性和稳定性，把改革创新贯穿于经济社会发展各个领域各个环节，以改革促发展、促转型、促民生。从省内来看，全省上下正在充分挖掘“过渡带”、“结合部”的叠加优势，围绕实现“两个加快”、“两个率先”的总任务，着力促进“三量齐升”，全面推进“四化两型”、“四个湖南”建设，大力推动转型发展、创新发展、统筹发展、可持续发展、和谐安全发展，努力加快全面建成小康社会步伐。

　　我市正处在可以大有作为加快发展的重要时期。全市上下凝心聚力谋发展、促发展，发展基础逐步夯实，发展后劲逐步增强，多数经济指标增速在全省排名靠前，后发赶超态势逐步显现。我们必须深刻把握“中国梦”、“小康梦”、“两型梦”、“崛起梦”的基本内涵，以全面建成小康社会为统揽，紧紧围绕建设丽都益阳这个目标，切实抓住和用好各种机遇，特别是要把十八届三中全会精神作为推进益阳全面深化改革的宣言书和动员令，准确领会全面深化改革的指导思想、目标任务、工作重点和优先顺序，凝聚改革共识，落实改革举措，强化改革保障，坚定信心，振奋精神，开拓进取，攻坚克难，努力推动全市经济社会又快又好发展。

　　2014年政府工作的总体要求是：以邓小平理论、“三个代表”重要思想和科学发展观为指导，深入贯彻落实党的十八大和十八届三中全会精神，始终突出加快发展这个主题，全面深化各类改革，不断扩大对外开放，强力推进两大会战，大力实施“双百工程”，切实深化“三访三化”，着力改善民生民利，推动全市经济社会发展取得新的进步。

　　2014年的奋斗目标和工作重点

　　2014年经济社会发展的主要预期目标是：生产总值增长10.5%以上，公共财政总收入增长12%以上，固定资产投资增长25%以上，规模工业增加值增长12.5%以上，社会消费品零售总额增长13%以上，城乡居民人均可支配收入增长10%以上，人口自然增长率、万元生产总值能耗、主要污染物排放量均控制在省定目标以内。

　　重点是抓好以下十个方面的工作：

　　（一）强力推进改革开放。深化重点领域改革。深入推进政府机构改革，大力推动简政放权，继续抓好行政审批制度改革，巩固市本级行政许可项目在全省市州中最少这一成果。深入推进“营改增”改革；抓好全口径财政预算；加强财政资金绩效评估管理；启动国库集中支付电子化管理试点工作，推进乡镇国库集中收付制度改革；进一步创新税收协控联管办法，完善税收保障机制。健全城乡发展一体化体制机制，加快推进农村金融综合改革，稳妥推进农民住房财产权、林权、土地承包经营权抵押、担保、转让工作，重点抓好沅江市农村产权交易平台建设试点；继续推进村级治理机制改革、农村土地和房屋确权颁证、涉农资金整合等工作；完善村级组织运转经费保障机制，逐步化解村级债务；积极稳妥推进农村土地流转，实现流转面积300万亩以上；全面放开城镇户口管理，撤除制约门槛，加快农民变市民步伐。持续深化水务改革，落实最严格水资源管理制度，创新农村饮水工程建设管理模式。深入推进医疗卫生体制改革，优化公共卫生资源配置；稳步推进公立医院改革。不断深化企事业单位工资收入分配制度改革，启动事业单位分类改革，严格执行国家和省机关事业单位工资和津贴补贴政策，进一步落实企业工资集体协商制度。提升对外开放水平。强力推进“大招商、招大商”，加大引进战略投资者力度，确保在引进央企、国内外500强、上市公司上取得重大突破；加大产业链招商力度，确保在实现产业集群发展上取得重大突破；加大园区招商力度，确保在打造特色园区、专业园区上取得重大突破；加大以商招商力度，确保在吸引益阳籍在外知名人士、经济能人回乡兴业上取得重大突破。实现全年内联引资形成固定资产投资300亿元以上，实际利用外资2亿美元以上。加大立项争资工作力度，确保全年争取资金165亿元以上。加强出口商品品牌建设，提高出口商品质量，进一步加强与友好城市的合作，提高区域交流合作水平，实现外经外贸稳定增长。

　　（二）强力推进工业立市。继续坚持把新型工业化作为后发赶超的第一推动力。大力扶持大中小企业发展。加快培育优势骨干企业，大力实施百亿元企业培育工程，继续支持非公有制经济健康发展，促进中小企业转型升级。注重扶持本土企业发展。全力推进企业上市，新增省重点上市后备企业5家以上；加强创业园、小微企业孵化基地建设，鼓励个体工商户转“企”提升，加快企业“规下”升“规上”的步子，确保全年新增规模工业企业80家以上。培育发展战略性新兴产业。做好先进装备制造、新能源、新材料、生物医药、电子信息等产业发展规划，大力实施500亿元产业培育工程。努力提升传统产业发展水平。加快食品加工、林纸板材、农业机械、建材、有色冶炼等传统产业的改造提升。全年完成工业技改投资360亿元以上，完成重点技改项目40个以上。着力提高企业自主创新能力。引导企业特别是高新技术企业加大技术研发力度，提高自主创新能力。争取国家级科技创新项目2项以上、省级科技创新项目30项以上，新增省级技术（工程）中心2个以上；新增高新技术企业10家以上，全市高新技术企业达到100家以上。

　　（三）强力推进农业稳市。加强优质农产品基地建设。稳定粮食生产，突出发展黑茶、蔬菜、竹木、苗木花卉、水产五大优势产业。粮食生产面积稳定在620万亩以上，确保蔬菜种植面积170万亩以上，新增茶园面积2万亩、改造低产茶园1万亩。加快推进高效生态林业建设，完成造林24.5万亩，完成森林抚育经营62.2万亩，提升20万亩苗木花卉基地效益。稳定生猪生产。发挥“中国淡水鱼都”品牌效应，大力发展名特优水产养殖。推进农产品精深加工。加大对农业产业化龙头企业的政策扶持力度，重点培育年销售收入过10亿元的农业产业化龙头企业2家，过5亿元的5家，力争全市农产品加工业销售收入达到520亿元，农产品加工转化率达到60%以上。提升农业科技和农产品质量安全水平。认真实施农业科技推广工程，力争全年完成病虫害专业化统防统治面积200万亩以上，推广测土配方施肥技术720万亩以上。加强农产品质量安全监管体系建设，实现主要农产品农药残留和药物残留检测合格率达99%，确保不发生重大农产品质量安全事故。加快筹建“国家黑茶产品质量监督检验中心”和“全国安化黑茶产业知名品牌创建示范区”。加强农村基础设施建设。进一步推进水利“十大工程”建设，抓好小型农田水利工程，完成水利建设投入20.2亿元，完成水利工程4.35万处。完成环洞庭湖基本农田整治项目建设。深入推进农业气象服务体系和农村气象灾害防御体系建设。全面加快农村光纤到村进度，确保行政村光纤覆盖率达98%。突出抓好农网升级改造和农村电网建设。

　　（四）强力推进三产兴市。大力发展现代服务业。加快金融服务、证券保险、信息和科技服务、现代物流、电子商务等生产性服务业，及健康、养老、休闲等生活性服务业发展。大力加强物流基础设施建设，加快建设物流园区。进一步建设一批电子商务平台，重点培育一批电子商务龙头企业。加快金融服务业发展和债券市场建设，实现全年新增贷款100亿元以上，债券发行有新的突破。改造提升传统服务业。推动家政、商贸、餐饮、邮政等传统服务业转型升级。推进农超对接、网上对接和直供直销。继续推进城乡农贸市场标准化改造。着力培育一个营业额过100亿元的中心商圈，培育一批营业额过30亿元的商业中心。加快发展文化旅游产业。做好全市旅游总体规划修编工作。推动重大文化旅游创意项目建设，积极开展等级景区和工农业旅游示范点创建工作，抓好羊舞岭国家遗址生态公园、明清古巷建筑群规划建设和保护工作，做好铁铺岭遗址项目的前期工作，推动文化创意产业发展。促进房地产业持续健康发展。加强房地产市场引导，强化房地产市场监管，稳步推进房地产业发展。

　　（五）强力推进生态美市。大力加强生态文明建设，努力将益阳打造成生态优美的宜居城市。扎实推进节能减排。完成能耗下降和主要污染物排放量削减省定目标。继续加强工业、建筑、交通、公共机构等领域的节能工作，推进企业节能工程，确保万元规模工业增加值能耗下降5%。加快推进太阳能、水能、风能、生物质能等新能源项目。大力发展循环经济，力争成功申报一批省级以上循环经济试点示范市（县）、示范园区、示范企业。大力加强生态建设和环境保护。巩固退耕还林、石漠化治理、湿地保护等生态工程，加强薄弱地区生态修复。巩固渔业秩序整治成果，严厉打击乱捕滥猎、乱采滥挖行为，保护生态资源。按时按质完成十大环保工程项目建设。继续推进兰溪河、志溪河综合整治工作。切实抓好重金属污染防治工作，坚决关停排放不达标的矿冶企业，严格抓好矿冶项目环评工作。加强大气污染防治，有效促进氮氧化物减排；全面推进农村连片环境综合整治，巩固农村清洁工程成果，进一步改善农村环境。全面推进两型示范建设。纵深推进两型示范创建，培育20个以上市级示范创建单位、10个以上省级示范创建单位、2个以上省级示范单位。大力推进益阳东部新区两型示范区建设，提高两型带动区域经济发展水平。

　　（六）强力推进两大会战和“双百工程”。强力推进交通建设大会战。完成交通建设投资100亿元以上。确保常安高速、安邵高速益阳段和S225安化段、资北干线建成通车，确保益南、益安高速实质性开工，确保益娄高速益阳段、G319益阳南线高速、东坪至梅城公路等项目顺利推进。完成农村公路改造和建设里程500公里。加快南茅运河航运建设工程和清水潭、泥湾千吨级码头扩建工程建设。全面推进危桥改造、渡口、渡改桥、客运站场等工程建设。切实抓好农村公路管理和养护。强力推进园区建设大会战。做大做强国家级益阳高新技术产业开发区，理顺管理体制，突出工业招商，抓实项目建设，打造核心增长极。全市园区完成基础设施建设投资100亿元，建成标准化厂房100万平方米，提高标准化厂房利用率。抓好园区投融资平台、技术创新平台等公共服务平台建设。围绕投资、税收、项目等重点指标，强化园区考核，推动园区加快发展。强力推进“双百工程”。对纳入“双百工程”的大事和项目，精心部署安排，确保完成年度计划。突出抓好东部产业园汽车零配件基地、中联重科沅江产业基地、益阳电子信息产业基地、湖南工艺美术创意设计基地、江南古城、茶马古道、顺德城、海吉星等一批重大项目建设，确保在带动产业和区域发展上取得新的成效。

　　（七）强力推进新型城镇化。抓好城镇规划。完成中心城区总体规划修改方案的报批。修订完善中心城区控制性详细规划。完成县城和重点镇控制性详规和修建性详规编制。抓好农民集中居住区规划建设，确保每个乡镇编制1－2个农民集中居住区建设详规。坚持规划的刚性原则，加强规划执法，加强对中心城区的山体水体保护，进一步加大控违拆违工作力度。科学规划和及时落实拆迁安置用地，稳步推进先安置后拆迁的征地拆迁模式。加强城镇建设。加大城镇基础设施建设投入，确保全市城镇基础设施建设完成投资80亿元以上。实施一批地下管网改造、公园绿地建设、公共交通提质等市政重点工程。尽快完成益阳大桥的前期工作，实现大桥主体工程施工。继续抓好中心城区主次干道的提质改造和美化、绿化、亮化工程，加快资江两岸建设步伐。加大省、市级卫生乡镇创建力度，确保每个区县（市）每年成功创建一个省、市级卫生乡镇。强化城镇管理。深入推进城市综合执法和城管进社区工作。加大城市管理数字化、信息化、智能化建设力度。规范物业管理服务。巩固国家卫生城市创建成果，着力构建城市管理长效机制。突出抓好全国文明城市和国家交通管理模范城市创建工作，积极创建和申报国家新能源示范城市、国家环保模范城市和生态市、国家园林城市和国家历史文化名城。

　　（八）强力推进民生改善。全力抓好为民办实事工作，确保全面完成省政府下达的为民办实事目标任务。切实增加城乡居民收入。多途径增加城乡居民收入，确保全市城镇居民人均可支配收入增长8%以上，农村居民人均可支配收入增长11%以上。全力扩大就业与再就业。全面完成省级创业型城市创建工作。坚持以创业带就业，实现全年新增经济实体2.5万家以上，新增创业人数3.5万人以上，带动就业人数7.7万人以上。全面落实就业创业优惠政策，重点做好大中专毕业生、农村富余劳动力、城镇低收入家庭失业人员三大群体的就业工作。进一步完善社会保障体系。加强人力资源和社会保障基层公共服务平台信息系统建设。继续推进统筹城乡居民医保工作，积极推进居民医保大病统筹，探索建立职工医疗保险门诊统筹制度。探索“五险统一征缴”制度，强化社保扩面征缴。完善城乡社会救助体系，突出抓好城乡低保和农村五保供养工作。完善医疗救助制度和临时救助制度。继续实施农村敬老院改扩（新）建工程。完善失地少地农民的社会保障。落实低收入群体用电、用水、用气、有线收视和就医等价费优惠政策。努力办好人民满意的教育。积极推进教育综合改革，重点抓好人才培养模式和教师管理体制改革。进一步普及学前教育。完成100所合格学校（含公办幼儿园）建设任务，启动100所标准化学校建设工作；加大中心城区学校建设力度。加强职业教育基础能力建设。继续支持高等院校发展。提高人民群众医疗保健水平。积极稳妥开展城乡居民大病保险试点工作。建立和完善医疗质量控制体系。继续加强公共卫生服务体系建设，提高公共卫生服务均等化水平。加快基层医疗机构标准化建设。加强妇幼卫生和中医药工作。落实重大传染病和慢性病防治规划，做好重大疾病防治工作，提升卫生应急处置能力。努力改善城乡居民住房条件。抓好廉租房、公租房建设及棚户区改造、农村危房改造等工作。加强保障性住房的分配和运营管理。抓好25个农民集中居住区建设。推进非公经济组织住房公积金制度建设。大力推进扶贫攻坚和移民开发工作。全年减少贫困人口4.5万人以上。抓好移民集中区产业发展和柘溪库区环库公路建设等工程，继续实施特困移民避险搬迁安置工作。统筹抓好其他各项工作。认真实施“十百千万人才工程”，努力健全人才引进、培养、使用的科学机制。加强文物保护工作。扎实推进花鼓振兴工程，积极申报益阳花鼓戏为国家非物质文化遗产保护项目。认真组织好第三届中国湖南（益阳）黑茶文化节。扎实推进爱国卫生运动。深入推进全民健身运动，组织参加全省第十二届运动会。坚持计划生育基本国策，促进人口长期均衡发展。加强国土和矿产资源管理，坚持节约集约用地，保障重点项目的用地需求。突出抓好民生价格调控监管，努力保持“米袋子”、“菜篮子”等重要商品价格基本稳定。继续加强工会和青年工作。切实保护妇女儿童权益。重视残疾人权益保护，加快实施残疾人“两个体系”建设。完善民兵预备役、后备力量建设机制，加强国防动员信息化建设，提高国防动员和后备力量建设质量。认真做好第三次经济普查工作。全面做好国家安全、人民防空、工商行政管理、外事侨务、档案、台办、科协、地方志、保密、民族宗教、地震、水文等工作。

　　（九）强力推进社会治理。加强精神文明和民主法制建设。加强思想道德教育。开展和谐社区创建和农村社区建设试点，全面推进“四位一体”村级组织建设。抓好村级组织换届选举工作。深化法治先进城市创建工作，加强法制教育，提高全民法律素质，增强各级领导干部法治能力。全面加强人民调解委员会规范化建设，继续抓好行业性、专业性、区域性调解组织建设。开通法律援助绿色通道，进一步扩大法律援助覆盖面。强化社区矫正工作。健全公共安全体系。全面落实安全生产责任制，深入开展“打非治违”专项行动和重点行业领域的安全生产专项整治，防止各类重大安全事故发生。强化食品药品安全监管，保障公众饮食、用药安全。健全应急处置机制，提升突发事件应急处置水平。始终突出治安防范，稳步推进警务改革，切实加强新巡防工程、社区警务和天网工程建设，推进平安医院、平安校园、平安小区创建工作，进一步强化公安基层基础。全力维护社会稳定。坚持系统治理、依法治理、综合治理和源头治理多种方式统筹推进，创新矛盾预防化解机制。深化社会稳定风险评估，继续推进维稳工作“村为主”模式，深入排查化解矛盾纠纷。改革信访工作方式，建立网上受理信访制度。加强网络信息监管和舆情应对，妥善处置网上炒作和网络群体性事件。

　　（十）强力推进“三访三化”。结合开展党的群众路线教育实践活动，紧紧围绕“干部受教育、群众得实惠、经济促发展、社会增和谐”的目标，进一步深化“三访三化促发展解民忧”专项活动。加强工作部署。将“三访三化”活动开展情况纳入年度工作计划，与经济社会发展的各项工作同安排、同考核。坚持统筹推进。强化各级各部门的协调配合，构建“上下联动、左右协调”的工作机制，形成市县乡三级、各部门单位协力推进的良好局面，实现全市上下“一盘棋”。突出工作重点。将活动的着力点放在改进工作作风、加强队伍建设上，放在帮助基层和群众解决实际问题上，放在促进经济发展和民生改善上，提高群众满意度。确保工作效果。强化督促检查，加强考核评估，严格奖优罚劣，激励创先争优，确保活动取得良好效果，推动活动向纵深发展。

　　优化发展环境和加强政府自身建设

　　大力优化发展环境。继续开展“发展环境优化年”活动。进一步优化政务环境。推进行政审批规范化、电子化，最大限度减少行政审批环节，优化审批程序，推行即时审批、限时审批，抓好行政处罚和工程招投标、产权交易、土地招拍挂、政府采购等公共资源市场化配置网上运行与电子监察。切实加强诚信建设，国家工作人员要带头讲诚信。进一步优化执法环境。加强行政执法规范化建设，认真执行行政处罚自由裁量权标准，促进公正文明执法。进一步优化企业环境。严格执行涉企检查“宁静日”、“备案制”、“公示制”、“报告制”等制度，认真清理涉企收费项目，切实减轻企业负担。进一步优化建设环境。依法打击强装强卸、强揽工程、强行阻工等违法犯罪行为。进一步优化政策环境。严格执行省市优化经济发展环境的有关政策规定，加强监督检查，集中治理办事难、审批慢、乱检查、乱收费、索拿卡要等问题，对行政不作为、乱作为等损害经济发展环境的违纪违规行为保持“零容忍”，发现一起，查处一起，决不姑息。

　　切实加强政府自身建设。加快转变政府职能。全面正确履行政府职能，加强政府公共服务、市场监管、社会管理、环境保护等职责，最大限度减少政府对微观事务的管理，充分发挥市场对资源配置的决定性作用。全面提高行政效能。切实改进文风、会风，政府系统规范性文件下发数量和全市性会议均减少30%以上，提倡开短会、讲短话。最大限度减少各类检查、评比和达标活动。加强政务公开，推进电子政务，简化审批流程，落实服务承诺，提高办事效率。切实规范政府行为。坚持科学、民主决策，建立决策问责和纠错制度。全面推进依法行政，认真落实人大及其常委会的决议、决定，主动接受人大及其常委会的监督，支持人民政协参政议政，认真听取民主党派、工商联、无党派人士的意见，认真办理人大代表建议、批评意见和政协委员提案，自觉接受群众监督、媒体监督。不断加强廉政建设。认真落实各级关于加强党风廉政建设和改进作风的有关规定，严格规范办公用房、公务用车和办公经费，推动财政预算公开和“三公”经费公开，实现“三公”经费零增长。坚持用制度管权管人管事，加强对权力运行的制约和监督，不断加大惩治和预防腐败工作力度，着力建设廉洁政府。

　　各位代表，当前益阳正处在奋力后发赶超、全面建成小康社会的关键时期，责任重大，使命光荣。让我们在中共益阳市委的坚强领导下，全面贯彻落实党的十八大和十八届三中全会精神，以更加昂扬的斗志、更加务实的作风，锐意进取，顽强拼搏，为加快益阳全面建成小康社会步伐而努力奋斗！
